# Assessment of Integrative Therapeutic Methods for Improving the Quality of Life and Functioning in Cancer Patients—A Systematic Review

**DOI:** 10.3390/jcm13051190

**Published:** 2024-02-20

**Authors:** Mădălina Gabriela Iliescu, Liliana-Elena Stanciu, Andreea-Bianca Uzun, Adelina-Elena Cristea, Irina Motoască, Laszlo Irsay, Dan Marcel Iliescu, Titus Vari, Alina Deniza Ciubean, Bogdan Marian Caraban, Nicolae Ciufu, Olgun Azis, Viorela Mihaela Ciortea

**Affiliations:** 1 Department of Physical Medicine and Rehabilitation, Faculty of Medicine, Ovidius University of Constanta, 1 University Alley, Campus—Corp B, 900470 Constanta, Romania; liliana.stanciu@365.univ-ovidius.ro (L.-E.S.); bianca.uzun@365.univ-ovidius.ro (A.-B.U.); adelina.ungureanu@365.univ-ovidius.ro (A.-E.C.); 2 Medical Doctoral School, Faculty of Medicine, Ovidius University of Constanta, 1 University Alley, Campus—Corp B, 900470 Constanta, Romania; 3 Department of Rehabilitation Medicine, University of Medicine and Pharmacy “Iuliu Hatieganu”, 8 Victor Babes Street, 400012 Cluj-Napoca, Romania; motoasca.irina@elearn.umfcluj.ro (I.M.); laszlo.irsay@umfcluj.ro (L.I.); titus.vari@elearn.umfcluj.ro (T.V.); alina.ciubean@umfcluj.ro (A.D.C.); viorela.ciortea@umfcluj.ro (V.M.C.); 4 Department of Anatomy, Faculty of Medicine, Ovidius University of Constanta, 1 University Alley, Campus—Corp B, 900470 Constanta, Romania; dan@anatomie.ro; 5 Department of Plastic Surgery, Faculty of Medicine, Ovidius University of Constanta, 1 University Alley, Campus—Corp B, 900470 Constanta, Romania; bogdan.caraban@365.univ-ovidius.ro; 6 Department of General Surgery, Faculty of Medicine, Ovidius University of Constanta, 1 University Alley, Campus—Corp B, 900470 Constanta, Romania; nicolae.ciufu@365.univ-ovidius.ro; 7 Department of Urology, Faculty of Medicine, Ovidius University of Constanta, 1 University Alley, Campus—Corp B, 900470 Constanta, Romania; olgun.azis@365.univ-ovidius.ro

**Keywords:** rehabilitation, cancer therapy, integrative, physical exercises, nanotechnologies, quality of life

## Abstract

**Background:** Cancer rehabilitation represents a series of measures adopted for the recovery of psychological, emotional, social, and financial functioning in the case of cancer patients. The purpose of this study is to identify the main elements of therapeutic management in the field of medical rehabilitation, as well as integrative, complementary medicine and holistic approaches that can be performed on the oncological patient. **Methods:** This systematic literature review follows the methodology outlined in the “Preferred Reporting Items for Systematic Reviews and Meta-Analysis” (“PRISMA”) statement, which is an internationally recognized and widely accepted standard. **Results:** Active rehabilitative therapies offer therapeutic options for improving the functioning and quality of life of oncological patients; these therapies comprehensively address both the physical and psychological aspects of the disease. This review also includes the latest novelties and nanotechnologies applied in oncological rehabilitation, for example, drugs (or supplements) inspired by nature. **Conclusions:** Physical and rehabilitation medicine, mostly using stimulating therapeutic methods, was recently added to the list of contraindications in the management of oncological patients, both as an approach to the pathological concept itself and as an approach to the main clinical consequences and functional aspects of oncological therapies. Integrative, complementary medicine presents an important therapeutic resource in the case of oncological patients. Advanced studies are needed in the future to further ascertain the role of these therapies.

## 1. Introduction

Integrated therapeutic methods to improve the quality of life among cancer patients represent an essential component of the holistic approach to the management of this disease. These methods focus on improving the patient’s well-being in all aspects of their life, including physical, emotional, and social aspects. The following are some of the integrated therapeutic methods that have shown significant benefits in this regard: meditation; yoga; specific nutrition and nanotechnologies inspired by nature; art and music therapy; counselling; acupuncture; and rehabilitation treatments including massage, exercise, electric current therapy/electrotherapy, and balneotherapy.

This systematic review aimed to analyze the effect of rehabilitation medicine and nanotechnologies—supplements inspired by nature that aim to improve the quality of life and functioning in cancer patients. The need to carry out this study is evident from the fact that not all methods of medical rehabilitation represent contraindications for this type of patient, regarding both the pathological concept itself and the main clinical consequences and functional aspects of the oncological therapies applied in various types of cancer, such as surgery, chemotherapy, immunotherapy, hormone therapy, and radiotherapy. Active rehabilitative therapies in oncorehabilitation are planned for deconditioning statuses, different types of pain, cardiorespiratory dysfunction, failure risk, walking problems, daily living abilities, disorders of the venous and lymphatic systems, skin troubles, etc., with the choice of therapy being directly related to the type of disability. The medical field of oncorehabilitation must be developed, and this type of study participates in this process.

Alternative therapies in the rehabilitation of cancer patients constitute a vast and constantly evolving field, with a holistic approach aiming to improve quality of life and support the healing process. While conventional treatments such as chemotherapy and radiotherapy remain the cornerstones of cancer management, alternative therapies offer a complementary perspective, bringing emotional, physical, and psychological benefits [1].

The rehabilitation of cancer patients is not limited to fighting tumors but also aims to restore the overall balance of the body, including the physical, emotional, and social aspects. Alternative therapies complement this integrative approach, helping to manage the adverse symptoms of conventional treatments and promoting a healthy lifestyle [1,2].

Acupuncture, massage, and physical therapeutic procedures including balneal treatments have been successfully used due to their ability to reduce the nausea and fatigue associated with chemotherapy and provide patients with the possibility to manage their pain, reduce anxiety, and relieve the side effects of cancer treatments [3,4].

Regular exercise is also essential for maintaining physical function and vitality [4,5]. A healthy and balanced diet plays a crucial role in supporting the immune system and improving the energy of cancer patients, even when combined with specific supplements inspired by nature. Consultation with a nutritionist can help in developing a meal plan tailored to the patient’s individual needs [3]. Artistic expression and interaction with art or music can have significant therapeutic effects and can serve as ways to express emotions, providing patients with a way to cope with stress and anxiety [3]. Counseling and psychosocial support services are essential in the integrated approach to cancer patients. They can provide a safe space for expressing emotions, managing anxiety, and developing coping strategies [3].

An important aspect of alternative therapies in the rehabilitation of cancer patients is complementary therapy, which is often used alongside traditional medical treatments [6]. In addition, alternative therapies are often associated with a personalized and patient-centered approach. By focusing on the individual needs of each patient, these therapies can provide significant benefits in managing the side effects of treatments, as well as supporting the recovery process.

Meditation and mindfulness practices have been associated with reduced levels of stress, anxiety, and depression among cancer patients. These techniques help patients improve their concentration and cope better with the stress of diagnosis and treatment [7]. Yoga offers physical as well as emotional benefits. Through a combination of movement, breathing, and relaxation, yoga can help reduce fatigue, improve flexibility, improve sleep quality, and reduce stress and anxiety, thus contributing to patients’ overall well-being.

## 2. Materials and Methods

This systematic literature review was designed using the methodology outlined in the Preferred Reporting Items for Systematic Reviews and Meta-Analysis (PRISMA) statement, an internationally recognized and widely accepted standard [8]. The protocol for this review was registered in PROSPERO (International Prospective Register of Systematic Reviews); protocol registration: CRD42023475518.

For the selection of eligible articles for our research, we searched the following databases: PubMed, Physiotherapy Evidence Database—PEDro, Cochrane and Elsevier. The following keywords were used to search the databases: physical exercises—cancer rehabilitation—quality of life, water-based physical exercises—cancer rehabilitation—quality of life, transcutaneous electrical nerve stimulation—cancer rehabilitation—quality of life, ultrasound—cancer rehabilitation—quality of life, laser—cancer rehabilitation—quality of life, extracorporeal shockwave therapy—cancer rehabilitation—quality of life, balneotherapy—cancer rehabilitation—quality of life, hypoxia–hyperoxia—cancer rehabilitation—quality of life, massage therapy—cancer rehabilitation—quality of life, cancer quality of life—rehabilitation—nanotechnology, cancer supplements—quality of life, lymphedema—cancer rehabilitation—quality of life (Table 1).

Articles were sorted by title and abstract using the following criteria for inclusion: articles written in English; articles no older than 10 years; cancer patients regardless of cancer type; cancer complications; rehabilitation; oncologic rehabilitation; quality of life; free full text; articles fall under the category of evidence-based medicine articles. The exclusion criteria were as follows: articles written in a language other than English; articles published more than 10 years ago; books and book chapters; conference abstracts. Citations from PubMed, Elsevier, Cochrane and PEDro were extracted and entered into Zotero. Later, all the resulting citations were exported to Excel and the duplicates were extracted using the “remove duplicates” function. The filters applied on PubMed were as follows: free full text; clinical trial; meta-analysis; randomized controlled trial; review; systematic review; written in the last 10 years.

The studies’ methodological quality—risk bias—was evaluated by two independent reviewers using the PEDro scale, a validated instrument for assessing the quality of clinical trials. All articles showed high quality, with it being noted that the nature of the intervention regarding exercises means that the participants and physiotherapists could not usually be blinded. Any disagreements were resolved by consulting a third reviewer if the first two reviewers could not reach an agreement.

The question on which this review paper was based, using the PICO (patient/problem, intervention, comparison and outcome) strategy, was as follows: *For oncologic patients, what are the effects of integrative therapeutic methods on functioning and quality of life compared with no intervention?*

## 3. Results

**Figure 1:**
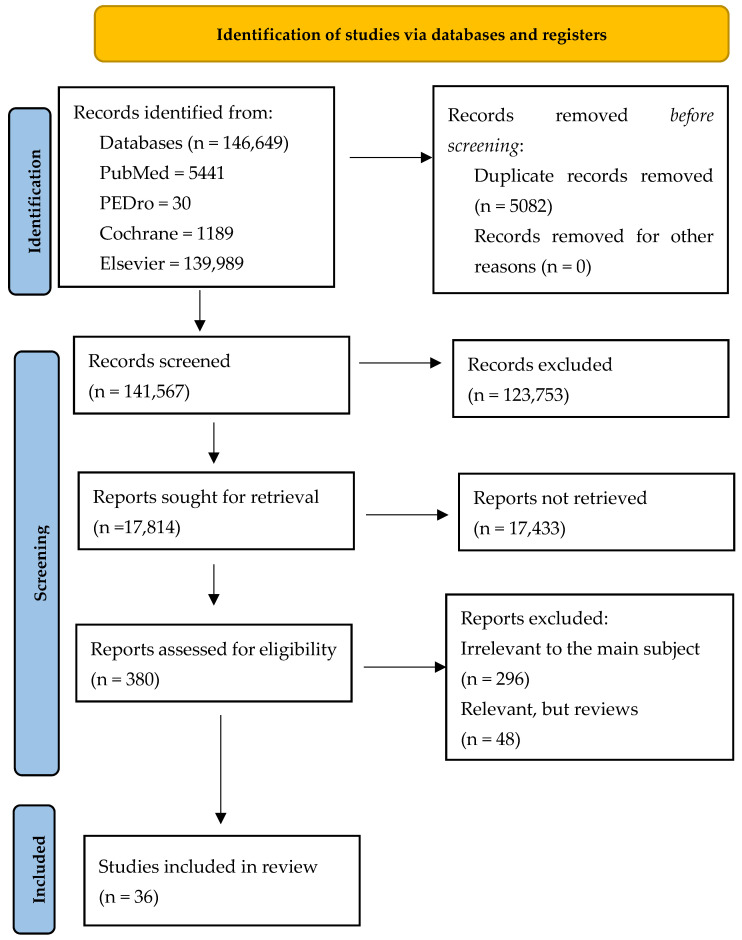
Our adapted PRISMA flow diagram.

The general search revealed 146,649 articles: 5441 in PubMed, 30 in PEDro, 1189 in Cochrane and 139,989 in Elsevier. We found 508 duplicate records. A total of 123,753 studies were excluded after screening the titles and the abstracts. From the remaining 380 papers, a selection was made and only the papers related to the subject were chosen, resulting in the selection of 84 articles, including reviews. After removing the reviews, a total of 36 articles were analyzed (Figure 1).

The resulting research articles (36) were listed in the table below, including authors, the year of publication, type of cancer (breast, prostate, head and neck, lung, gastrointestinal, gynecologic, spinal bone metastases or clinical conditions after oncological treatments), therapeutic intervention, type of monitoring and conclusions (Table 2).

The data obtained from the articles were analyzed based on the main research theme; the synthesis of the studies is presented below.

### 3.1. Land- and Water-Based Physical Exercises in Cancer Patients

Dieli-Conwright et al. (2018) evaluated 100 breast cancer survivors engaging in moderate-to-vigorous aerobic and resistance exercises three times a week for a duration of 16 weeks. The exercise intervention led to significant improvements in various aspects of their lives, including quality of life, reduction in depression, decreased fatigue, and enhanced physical fitness. These positive changes were sustained throughout the three-month follow-up period [25].

Another randomized controlled trial (RCT) involving 148 patients undergoing chemo-radiotherapy for head and neck cancer aimed to evaluate the effectiveness of physical exercises in improving functional capacity. The control group was provided with recommendations to engage in three ten-minute walks per day, five days a week, while the exercise group received a structured exercise intervention consisting of both aerobic and dynamic resistance exercises, with a frequency of five times a week. The comprehensive exercise-based rehabilitation approach yielded positive outcomes in terms of improved functioning, enhanced quality of life, and reduced levels of fatigue among the patients [20].

The purpose of a review conducted by Schmidt et al. in 2014 was to investigate whether incorporating resistance exercises during chemotherapy had a positive effect on fatigue levels and quality of life in breast cancer patients who had just started chemotherapy. The results provide evidence of significant and clinically meaningful benefits associated with resistance exercise in terms of reducing fatigue and enhancing quality of life during chemotherapy [42].

In 2019, Steindorf et al. demonstrated the potential of resistance training as a promising approach to alleviate symptoms, enhance physical function, and improve the overall quality of life for individuals dealing with pancreatic cancer. The SUPPORT trial (Supervised Progressive Resistance Training for Pancreatic Cancer Patients) was designed as a three-arm study, comprising a supervised progressive resistance training group, a home-based progressive resistance training group, and a control group; patients in the resistance training groups engaged in sixty-minute exercise sessions twice a week over the course of six months [21].

Endurance and balance training exercises offer tangible and meaningful advantages for cancer patients who continue to experience chemotherapy-induced peripheral neuropathy. It has been shown that endurance training can alleviate sensory symptoms, while balance training improves the functional status of patients [24].

A total of one hundred and sixty breast cancer patients ranging from stage 0 to III were randomly allocated to either a twelve-week progressive resistance training program (involving machine-based resistance exercises, lasting sixty minutes and conducted twice a week) or a twelve-week relaxation control group (consisting of muscle relaxation exercises, also lasting sixty minutes and held twice a week); the progressive resistance training program proved to be a safe, viable, and effective strategy for improving the quality of life and reducing fatigue in breast cancer patients undergoing adjuvant radiotherapy [41].

Personal training sessions that lasted a maximum of thirty weeks, with each session involving three individual exercises conducted under the guidance of a certified oncology fitness trainer, led to noteworthy enhancements in terms of endurance and strength but showed no statistically significant improvements in physical functioning or quality of life, as reported by Wang et al. in 2021 [16].

Moderate-intensity physical activity improved functional capacity, reduced levels of inflammatory biomarkers and fatigue, and had a positive impact on the quality of life for prostate cancer patients undergoing radiotherapy, compared to control, as shown in a cohort of 54 prostate cancer patients at high risk [29].

Another large study, the PROSPER trial, a multicenter randomized controlled trial conducted across 17 National Health Service cancer facilities in the UK, involved 392 women who were undergoing surgery for breast cancer and were at risk of developing postoperative complications in the upper limb; the patients were randomly assigned in a 1:1 ratio to either receive standard care along with a physical exercise program (comprising strengthening exercises, physical activity, stretching, and behavioral change techniques) starting 7 to 10 days after surgery or to the standard care group. The assessment of upper extremity function was performed using the DASH questionnaire one year after the randomization. The study findings indicated that implementing a physiotherapy exercise regimen within the first 7 to 10 days after breast cancer surgery was both clinically effective and cost-effective. This approach reduced upper limb disability in breast cancer patients who were at risk of postoperative complications, and it did not lead to an increase in wound-related issues, neuropathic pain, or lymphedema symptoms one year after the intervention [13].

A 2018 study conducted by Mijwel et al. revealed that engaging in 16 weeks of resistance and high-intensity interval training is effective in preventing the worsening of cancer-related fatigue and reducing the overall symptom burden experienced by breast cancer patients undergoing chemotherapy [30]. Another study, in 2020, by Hong et al. showed that machine-based resistance exercise training positively contributed to reducing the occurrence of symptoms such as nausea and acid reflux, enhancing physical function, and alleviating fatigue and loss of appetite in patients with gastrointestinal cancer undergoing chemotherapy [18].

A clinical trial conducted on 60 patients with spinal bone metastases who were undergoing radiotherapy compared the impact of resistance training and passive physiotherapy on the quality of life, levels of fatigue, and emotional distress. The results indicated that isometric resistance training targeting the paravertebral muscles led to improvements in the quality of life, reductions in fatigue, and enhancements in functional capacity, alongside alleviating the specific concerns related to the fear of losing mobility and becoming dependent on others [40]. Additionally, a combination of aerobic, resistance, and flexibility exercises in patients with head and neck cancer undergoing chemotherapy yielded positive benefits in terms of physical fitness and health-related quality of life, as demonstrated by Lin et al. (2021) [15].

The UMBRELLA Fit study involved 260 breast cancer patients who were randomly divided into an intervention group that received 12 weeks of supervised exercises consisting of 60 min of aerobic and resistance training, and a control group that received standard care; since only half of the patients chose to participate in the exercise intervention, the exercise program did not have a significant impact on the patients’ quality of life, with only a minor improvement in fatigue levels observed [14].

The objective of another trial conducted in 2014 was to investigate the effects of various types and durations of exercise on 301 breast cancer patients undergoing chemotherapy; one group engaged in standard-dose aerobic exercise lasting 25–30 min, another group performed a higher dose of aerobic exercises (50–60 min), and the third group conducted a combination of higher dose aerobic and resistance exercises (also 50–60 min). The study’s findings revealed that breast cancer patients who were in the premenopausal phase, younger, had a lower body mass index (BMI) and were undergoing chemotherapy were the most likely subgroup to experience improvements when engaging in higher-dose exercise routines [39]. Another study, including 204 breast cancer patients, showed that 18 weeks of exercise had a positive impact, resulting in benefits such as reduced fatigue levels, improved muscle strength, and enhanced cardiorespiratory fitness; however, by the 36-week mark after the intervention, these benefits were no longer statistically significant [36].

In a complex study, a total of 163 prostate cancer patients undergoing androgen deprivation therapy were evaluated during three exercise regimes: (1) supervised impact loading and resistance exercises for a duration of 12 months; (2) supervised aerobic and resistance training for 6 months, followed by a 6-month at-home exercise program; and (3) usual care group, where patients received printed booklets containing exercise information. Fatigue levels decreased in the group undertaking impact loading and resistance exercises both at 6 and 12 months after the intervention, while in the other two groups, the reduction in fatigue levels was observed only at the 12-month mark post-intervention [32].

There is also another older yet significant study by Lee et al., published in 2010, that evaluated 32 breast cancer patients divided into two groups; one group received scapula-oriented exercises, while the other group engaged in general exercises. Additionally, there was a third group consisting of 18 breast cancer survivors who did not receive any exercise treatment. The results indicated that scapula-oriented exercises led to improvements in pain management, quality of life, and strength. These exercises were particularly beneficial for enhancing quality of life and improving shoulder strength and range of motion [45]. The impact of rehabilitation on fatigue, quality of life, and cardiopulmonary function in breast cancer patients was also evaluated by Do et al. in 2015. Upon completing the multimodal rehabilitation program, the study revealed that patients who underwent this intervention experienced notable improvements in their quality of life, reductions in fatigue levels, and the alleviation of physical symptoms [35].

A study conducted by Odynets et al. in 2019 evaluated the effects of water exercises, Pilates or yoga on different aspects of quality of life in 115 breast cancer patients; the intervention period was 1 year, summarized in 144 rehabilitation sessions. Results showed a significant increase in quality-of-life indicators for the water exercise group, especially for the emotional well-being item. It was found that water exercises are more effective for decreasing negative symptoms associated with cancer treatment than Pilates or yoga [23]. Another systematic review conducted by Wang et al. in 2022, which included 356 participants, showed that aquatic physical therapy interventions significantly reduced fatigue compared with standard care; additionally, compared with land-based training, aquatic physical therapy improved the quality of life, even though it did not improve the physical index (waist circumference) compared to usual care [46].

### 3.2. Massage Therapy and Cancer

Results show that massage therapy could be a useful intervention for reducing pain in children with cancer, more effective in decreasing the interference of pain during ambulation in a randomized, controlled, single-blind trial conducted by Batalha et al. in 2013 on 52 children aged 10 to 18 years who were hospitalized in a pediatric cancer ward; the intervention consisted of a massage protocol with three sessions on alternate days over a one-week period [47].

A systematic review carried out by Boyd et al. (2016), which included 12 high-quality and 4 low-quality studies, demonstrated that massage therapy is effective for treating pain, fatigue and anxiety compared to the active comparators (e.g., attention, usual care, standard treatment, a reading group comparator, caring presence). Focused only on the subgroup of cancer pain populations, the authors concluded that massage therapy should be considered as a therapeutic option to help manage their cancer-related pain, while their evidence synthesis could not be conducted on health-related quality of life, emotional stress, and activity outcomes due to insufficient research [48].

Anma massage therapy (also known as Japanese massage therapy, AMT) is a popular complementary and alternative medicine treatment in Japan. According to anecdotal evidence, it has long been used to relieve physical and psychological complaints in both healthy and cancer-related individuals. Donoyama et al. (2018) examined 40 gynecologic cancer survivors receiving either a 40-min AMT session per week for 8 weeks or no AMT. Quality of life was assessed and scores on fatigue and insomnia showed significant improvement in the AMT group, while no improvement in anxiety and depression scales was found [26].

A randomized early-phase trial was conducted on 66 female survivors of breast cancer to assess the effects of Swedish massage therapy (SMT) vs. light touch (LT) or waitlist control (WLC) on CRF. It was found that SMT produced clinically significant relief from CRF, being superior to LT or WLC [33].

Another pilot randomized controlled trial investigated a protocol of Swedish massage therapy for symptomatic relief of chemotherapy-induced peripheral neuropathy (CIPN) to determine the ideal weekly frequency and number of weeks of providing massage; the results indicate that offering two distinct oncology massage programs and timetables to cancer survivors impacted by CIPN is highly feasible. With no variations in adherence, giving massages three times a week for four weeks tended to produce better results than giving them twice a week for six weeks [12].

In 2016, a Cochrane systematic review was conducted on the effects of massage with or without aromatherapy for symptom relief in people with cancer; it included 21 reports of very low-quality evidence, including a total of 1274 participants. Furthermore, this review demonstrated no differences in the effects of massage on depression, mood disturbance, psychological distress, nausea, fatigue, physical symptom distress, or quality of life when compared with no massage, and no evidence supporting the use of this intervention for clinical benefit was found [49].

### 3.3. Nanotechnologies Based on Natural Products for Cancer Patients

Medicinal plants with specific nanotechnologies contain a variety of phytochemicals and bioactive compounds including carotenoids, anthocyanins, resveratrol, flavonoids, polyphenols, caffeic acid, epigallocatechin, saponins, quercetin, luteolin, rosmarinic acid, carnosic acid, and more. These phytochemicals are sourced from different parts of plants including the seeds, fruits, roots, and leaves. They can induce apoptosis through the mitochondrial pathway. This process involves the release of cytochrome C and/or caspase activation. They contribute to the generation of an apoptosis-inducing factor (AIF). The bioactive compounds found in plants can influence various stages of carcinogenesis, potentially inhibiting, delaying, or reversing the progression of tumors before they become invasive malignancies [50].

Considerable evidence has demonstrated the potential advantages of rosmarinic acid (RA) and RA-enriched plants as promising candidates for cancer prevention and treatment. RA-rich plant varieties, such as rosemary, basil, and Perilla frutescens, have the potential to act as anti-tumor agents when used as dietary supplements. The antioxidative and anti-inflammatory effects of RA play a role in preventing tumorigenesis, suggesting that oral administration of RA could serve as a potential approach to prevent colorectal cancer (CRC). RA exhibits anti-tumor properties by impeding tumor cell proliferation and epithelial–mesenchymal transition (EMT), causing cell cycle arrest and promoting apoptosis. This process involves various pathways, including PI3K/AKT (Protein kinase B), necrosis factor—NF-κB, interleukin—IL-6/STAT3 (Activator of transcription 3), p53, vasculo—endothelial grow factor—VEGF, and glycolysis. The inhibition of multidrug resistance (MDR) protein by RA enhances chemosensitivity in tumor therapy. RA is extensively utilized in treating digestive system tumors, particularly hepatocellular carcinoma (HCC) and CRC. Additionally, RA has the potential to heighten the sensitivity of cisplatin (DDP) and doxorubicin (DOX) drugs in the treatment of solid tumors. To enhance the oral bioavailability of RA, promising approaches include modifying excipients, employing cyclodextrin encapsulation, utilizing drug delivery systems, and developing RA derivatives [51].

Carotenoids are organic, liposoluble pigments distributed in vegetables, colored fruits, photosynthetic bacteria, fungi, algae, and some fish. There is great interest in natural compounds utilized in managing various symptoms and illnesses in the present era. Individuals undergoing palliative care due to challenging symptoms linked to advanced-stage cancers and ineligible for anti-cancer treatments are interested in utilizing dietary supplements and herbal remedies to potentially improve their prognosis, reduce their symptom severity, and improve their quality of life. Carotenoids represent a group of natural chemical compounds showing remarkable promise, primarily attributed to their strong antioxidant, anti-inflammatory, and neuroprotective properties. Over centuries, these compounds have been a part of folk medicine, utilized to treat a multitude of diseases and alleviate their associated symptoms [52].

Ginger, a traditional remedy used by many cultures for gastrointestinal issues, has been supported by previous clinical trials for its efficacy in alleviating nausea, particularly in contexts such as chemotherapy-induced nausea and vomiting (CINV). In two previous clinical trials, ginger supplementation showed comparable effectiveness to metoclopramide in reducing CINV symptoms. Both animal and cell culture studies have demonstrated a plausible mechanism for its anti-nausea effects. Animal studies have preliminarily suggested the potential role of ginger supplementation in preventing cisplatin-induced emesis [31].

The authors of a study carried out in 2017 hypothesized that supplementing with ginger could be an effective adjuvant treatment for chemotherapy-induced nausea (CIN). The study involved 51 patients who were randomly assigned to receive either 1.2 g of standardized ginger extract or a placebo daily. This supplementation was in addition to the standard anti-emetic therapy, and it was administered during the first three cycles of chemotherapy. Nausea was more prevalent than vomiting over three consecutive cycles of chemotherapy. During the first cycle of chemotherapy, participants in the intervention group reported significantly improved quality of life (QoL) related to CIN, chemotherapy-induced nausea, and vomiting (CINV)-related QoL as well as global QoL, and experienced less fatigue (*p* = 0.006) compared to those in the placebo group. There were no significant differences observed during the second cycle. In the third cycle, the intervention group had significantly improved global QoL and less fatigue compared to the placebo group. This trial suggests that supplementing with ginger as an adjuvant is associated with an improved chemotherapy-induced nausea-related quality of life and reduced cancer-related fatigue. There were no notable differences in adverse effects between the ginger-supplemented group and the placebo group [53].

A review carried out in 2020 on the use of green tea (*Camellia sinensis*) for the prevention of cancer concluded that results from epidemiological studies have presented inconsistent outcomes regarding the impact of green tea consumption on cancer risk, although some indications suggest a favorable effect on specific types of cancer. Moreover, most studies included were conducted within Asian populations known for their high green tea consumption, thereby restricting the applicability of these findings to other ethnic groups. The epidemiological evidence remains insufficient to support the advantageous role of green tea in reducing cancer risk. Additionally, the potential adverse effects associated with a high intake of green tea extracts should be carefully considered [54].

The intake of green tea has been associated with a decreased risk of breast cancer. A study conducted by Samavat et al. explored the impact of decaffeinated green tea extract (GTE) on circulating sex hormones and insulin-like growth factor (IGF) proteins. The study involved 538 healthy postmenopausal women who were randomly assigned to the GTE group and the placebo group; those in the GTE group took four decaffeinated capsules daily, containing 1315 mg total catechins (including 843 mg epigallocatechin-3-gallate) for one year; the placebo group ingested similar capsules lacking tea catechins. Blood sex hormones and IGF proteins (IGF-1 and IGF binding protein-3) were measured at the start and at 6 (for IGF proteins only) and 12 months. After 12 months, women in the GTE group had significantly higher total estradiol and bioavailable estradiol levels compared to those in the placebo group. A significant interaction between GTE supplementation and duration of treatment on estradiol levels and bioavailable estradiol was observed. The catechol-O-methyltransferase genotype did not impact blood sex hormones before or after GTE supplementation. The levels of circulating concentrations of IGF proteins were similar between the GTE and placebo groups at all three measurement points. These results indicate that a 12-month GTE supplementation notably increases circulating estradiol concentrations in the studied population (healthy postmenopausal women) [55].

A limited number of clinical trials involving bovine colostrum (BC) have illustrated its potential anti-cancer effects in different types of cancer. Numerous studies examining anti-cancer properties using BC supplements have been performed on rodent models, specifically treating rats and mice affected with colorectal, lung, and esophageal cancers using lactoferrin and conjugated linolenic acid (CLA). Preclinical trials reported a reduction in colon tumor burden and a decrease in VEGF expression. The exploration of BC’s anti-cancer potential in humans has thus far been limited, with only a small number of clinical trials conducted on a few patients. A clinical investigation involving 24 breast cancer patients administered CLA orally at 7.5 g/day for 20 days revealed inhibition in the expression of fatty acid synthase (FASN) and lipoprotein lipase (LPL), indicating breast tumor growth suppression. Another clinical study suggested the potential benefits of CLA (at 3 g/day) for rectal cancer patients undergoing chemoradiation. BC supplements have demonstrated efficacy in managing various human cancer cell lines such as esophageal, colorectal, lung, breast, and ovarian cancer. Specific constituents of BC, such as lactoferrin, CLA, and alpha-lactalbumin, have shown effectiveness in treating specific types of cancer. While BC has demonstrated anti-tumor properties in limited in vitro and animal studies, numerous BC components have shown the ability to induce apoptosis in cancer cells and limit tumor growth [56].

A study conducted in 2019 evaluated the safety and tolerability of curcumin combined with FOLFOX chemotherapy in patients with metastatic colorectal cancer, as well as the potential for any clinical benefit. A total of 28 patients, all over 18 years old, diagnosed with metastatic colorectal cancer were randomly divided into two groups; one group received folinic acid/5-fluorouracil/oxaliplatin chemotherapy (FOLFOX), while the other group received FOLFOX combined with 2 g of oral curcumin daily (CUFOX). The primary goal was to determine the safety and tolerance of adding oral curcumin to FOLFOX chemotherapy. Both groups presented similar adverse events. The combination of curcumin with FOLFOX chemotherapy proved to be a safe and tolerable treatment, offering potential benefits for patients [28].

*Actaea racemosa* L., commonly referred to as black cohosh, is a popular herbal remedy for alleviating menopausal symptoms. Due to its perceived estrogen-like activity, the extract from the root of black cohosh (BCE) has raised concerns about potentially stimulating the growth of breast cancer. To examine the impact of standardized BCE and its primary component actein on the growth rates of cells and the metabolism of steroid hormones, researchers investigated their effects on estrogen receptor alpha positive (ERα+) MCF-7 and estrogen receptor-negative (ERα-) MDA-MB-231 human breast cancer cells. The results indicated that actein and BCE neither encourage the growth of breast cancer cells nor influence estrogen levels. They stimulated androgen formation, which could contribute to alleviating menopausal symptoms in women. Since BCE led to an increase in androgen levels without affecting estrogen levels, it can be considered safe and does not promote the progression of tumors in patients with hormone-dependent breast cancer [57].

Phosphorylated inositol hexaphosphate (IP6), a naturally occurring carbohydrate, and its precursor compound myo-inositol (Ins) are prevalent in plants, particularly in high-fiber diets. Initial experiments on colon cancer conducted 30 years ago revealed that IP6 reduces cell proliferation, induces apoptosis and differentiation in malignant cells, leading to a return to a normal phenotype, and affects critical molecular targets. The enhanced immunity and antioxidant properties of IP6 also contribute to the destruction of tumor cells. While Ins has modest anticancer potential, the most significant anticancer outcomes have been observed with the combination of IP6 + Ins. Available as a dietary supplement, IP6 + Ins has demonstrated its ability to enhance the anticancer effects of conventional chemotherapy, control cancer metastases, and improve the quality of life for cancer patients. Ongoing clinical data and a vast amount of experimental data suggest its potential role either as an adjuvant or as an “alternative” to current cancer chemotherapy [58].

A double-blind randomized controlled trial involving 364 participants assessed the effectiveness and toxicity of a daily dose of 2000 mg of American ginseng for cancer-related fatigue. The evaluation utilized the Multidimensional Fatigue Symptom Inventory-Short Form (MFSI-SF), revealing a statistically significant enhancement in the fatigue score within the ginseng group compared to the placebo group after 8 weeks; participants concurrently receiving both ginseng and conventional cancer treatment demonstrated a statistically significant improvement in fatigue scores at both the 4th and 8th weeks compared to the placebo group. Another study explored the concomitant use of fermented Korean red ginseng extract in patients with non-small-cell lung cancer. A total of 34 patients were administered gemcitabine with cisplatin and fermented Korean red ginseng at 3000 mg daily, while 26 patients received only the chemotherapy drugs for 60 days. The study demonstrated a significant improvement in fatigue scores, cancer-related symptoms, psychological status, physical conditions, quality of life, and chemotherapy-induced adverse effects in the treatment group compared to the chemotherapy-alone group. No significant changes were observed in tumor markers when comparing the treatment group with the group receiving chemotherapy alone [59].

In 2015, Yan Han et al. conducted a study that aimed to evaluate the efficacy and safety of using Chinese herbal medicine (CHM) for patients with advanced non-small-cell lung cancer after conventional platinum-based first-line chemotherapy. A total of 106 patients were eligible and were randomly divided into two groups, with 99 patients completing the study. The patients in the study group received CHM every day until the disease became aggravated and the patients resigned or had unacceptable toxicity. Both groups received best-supporting care (BSC). Patients in the control group were treated with BSC recommended by the NCCN (National Comprehensive Cancer Network) Cancer Palliative Care Guide. Localized radiation therapy for pain relief was allowed, provided the radiation dose was in the palliative range. No other anticancer therapies were allowed during the study. Patients in the trial group received treatment consisting of both BSC and CHM. The CHM treatment involved the administration of a decoction containing 10–20 varieties of herbs. This herbal mixture was taken orally, 200 mL per administration, twice daily. Following treatment and the follow-up period for progression-free survival (PFS), no significant differences were observed. For quality of life (QOL), there were significant differences between the two groups as follows: physical well-being, emotional well-being, functional well-being, the lung cancer symptom domain, and the total score of the Functional Assessment of Cancer Therapy—FACT-L4.0. No substantial differences were found in the social well-being domain. No severe adverse effects related to the treatment were noted. The CHM was well tolerated, suggesting its potential to improve the quality of life in patients with NSCLC (non-small-cell lung cancer) [38].

A study conducted in 2016 aimed to determine the safety, maximum tolerated dose (MTD), and preliminary efficacy of saw palmetto (SP) for the management of lower urinary tract symptoms (LUTS) during radiation therapy (RT) for prostate cancer. Men with prostate cancer were recruited and received RT for eight weeks, five days a week; SP was started two weeks before RT started; patients were instructed to continue SP for two weeks after completion of RT. The dose-finding phase focused on determining the appropriate dosage and assessed the safety of three different doses (320, 640, and 960 mg) of SP. The randomized controlled trial phase aimed to evaluate the initial efficacy of the MTD compared to a placebo; in the dose-finding phase, 27 men completed the study without reporting any dose-limiting toxicities, with 20 participants reaching an MTD of 960 mg daily. In the exploratory randomized controlled trial phase, involving 21 men, no statistically significant differences were observed in the International Prostate Symptom Score. The prostate-specific concerns score of the Functional Assessment of Cancer Therapy- Prostate was higher in the SP group (*p* = 0.03). Although SP at 960 mg appears to be a safe herbal supplement, further investigation is needed to determine its efficacy in managing LUTS during radiotherapy [34].

Patients diagnosed with histologically confirmed squamous cell carcinoma of the head and neck, eligible for primary or adjuvant curative radiotherapy, with or without systemic treatment, were enrolled in a study that aimed to demonstrate that echium oil does not protect weight loss in patients undergoing curative radio(chemo)therapy. Participants in the intervention group (I group) consumed 7.5 mL bis in die (b.i.d., twice a day) (a total of 15 mL) of echium oil daily, starting from the initiation of treatment until the completion of the 7-week treatment period. Those in the control group (C group) received a placebo. In this study, echium oil supplementation proved to be feasible and well-tolerated, and it led to the anticipated increase in erythrocyte n-3 eicosapentaenoic acid (EPA) and dihomo-γ-linolenic acid (DGLA), without a significant increase in arachidonic acid (AA). Echium oil did not demonstrate a protective effect against weight loss induced by radio(chemo)therapy [43].

### 3.4. Intervention on Lymphedema

In 2015, Tomasz Gradalsk et al. conducted a study to compare the effectiveness of reducing postmastectomy arm lymphedema through two approaches: compression bandaging (CB) combined with physical exercises versus the same management enhanced by an additional 30 min of manual lymph drainage (MLD). A total of 60 patients were randomly assigned to the experimental CB group (CB-G) or the control CDT group (CDT-G). A total of 51 postmastectomy women participated in the study, which lasted 26 weeks, consisting of a 2-week intensive phase followed by a 6-month maintenance phase. Patients in the CB-G underwent treatment with multilayer compression bandages in addition to a standardized program involving physically active-assisted exercises combined with deep diaphragmatic breathing. The exercise sessions were conducted for 15 min once a day. Patients in the CDT-G received the same treatment as the CB-G, with the added component of 30 min of MLD based on the Vodder II method before the application of compression bandages. Deep diaphragmatic breathing was performed during the MLD sessions. Both groups underwent a two-week treatment, from Monday to Friday, with a break over the weekend when participants wore bandages. Throughout the six-month maintenance phase, all patients in both groups were advised to manage their weight, avoid limb overheating, protect the affected limb from accidental trauma, use gentle soaps for skin cleaning, and systematically moisturize the skin. Patients in both groups were instructed to continue the same program they followed during the intensive phase. The study results support the idea that compression bandaging combined with physical exercise can be considered a fundamental treatment option for limb lymphedema. The findings suggest that Vodder manual lymph drainage may not be necessary to achieve a comparable reduction in limb edema. These results indicate that both immediate and delayed effects can be achieved without using Vodder manual lymph drainage, and compression bandaging may be an essential part of lymphedema management [37].

An article from 2015 proposed evaluating the efficacy and safety of manual lymphatic drainage (MLD) in treating breast-cancer-related lymphedema (BCRL). In this study, the authors included six trials, clustered into three categories. First category: MLD combined with standard physiotherapy, compared to standard physiotherapy alone in a single trial, demonstrated notable improvements in both groups from the initial assessment. However, there were no statistically significant differences between the groups in terms of percent reduction. Second category: in two trials comparing MLD combined with compression bandaging versus compression bandaging, notable percentage reductions of 30% to 38.6% were observed for the group using compression bandaging alone, and for MLD resulted in an additional 7.11% reduction; volume reduction was borderline significant. Subgroup analyses were significant and indicated that participants with mild-to-moderate BCRL were more responsive to MLD compared to those with moderate-to-severe BCRL. Third category: the results from three trials comparing MLD combined with compression therapy versus non-MLD treatment with compression therapy were too diverse to be pooled. In one trial, the comparison involved a compression sleeve plus MLD versus a compression sleeve plus a pneumatic pump. The findings indicated a statistically significant volume reduction favoring MLD; the percent reduction was borderline, and the changes in lymphedema (LE) volume were non-significant. Another trial compared a compression sleeve plus MLD to a compression sleeve plus self-administered simple lymphatic drainage (SLD), revealing significance for MLD in terms of LE volume but not for volume reduction or percent reduction. The third trial, comparing MLD plus compression bandaging versus SLD plus compression bandaging, did not show any significance for percent reduction, the only outcome measured [60].

Xiaoli Wu et al. conducted a study to investigate the efficacy of early prevention using complex decongestive therapy (CDT) and rehabilitation exercises for the prevention of postoperative lower limb lymphedema in patients with gynecologic cancer. This prospective randomized controlled study included a total of 109 female patients who had undergone surgery for cervical cancer, endometrial, or ovarian cancer. The patients were randomly assigned to two groups: the control group, which received routine treatment only, and the CDT group, which underwent both CDT and rehabilitation exercises. The study recorded the incidence of lower extremity lymphedema, utilizing a designed scale for assessing patients’ lower extremity lymphedema and measuring the diameter of the lower limbs. Quality of life was measured using the European Organization for Research and Treatment of Cancer Quality of Life Questionnaire (EORTC QLQ-C30) and the Brief Fatigue Inventory (BFI). The results indicated that early prevention with CDT combined with rehabilitation exercises led to a reduced incidence of lower limb extremity lymphedema. Furthermore, this approach improved patients’ quality of life and contributed to a reduction in cancer-related fatigue [19].

The physical therapy treatment for lymphedema patients includes treatment based on the principles of complete decongestive therapy (CDT), which consists of skin care, manual lymphatic drainage, bandaging, and exercises. A protocol study proposed by M. Tambour aims to explore whether CDT is equally effective with or without the inclusion of manual lymphatic drainage in the treatment of arm lymphedema in patients with breast cancer. The trial design is as follows: a total of 160 breast cancer patients with arm lymphedema will be randomized into two treatment groups. The patients from Group A will receive CDT including manual drainage, skin care, bandaging using Coban™2 Lite, and guidance on physical activity. The patients will undergo manual lymphatic drainage sessions for 30 min twice a week. The total treatment duration of each CDT treatment session will be one hour. The patients from Group B will receive skincare, bandaging using Coban™2Lite, guidance on physical activity, and CDT without manual lymphatic drainage. Each treatment session will last for 30 min and will be administered twice a week. The intervention will span approximately 4 weeks, followed by a 6-month follow-up period (7 months from baseline). Standardized measurements will be conducted before randomization, after 4 weeks, and again after 7 months. This randomized controlled study aims to provide information on an effective treatment for patients with breast-cancer-related arm lymphedema, while simultaneously minimizing inconvenience for the patients [61].

In 2020, M. N. Muñoz-Alcaraz et al. proposed a protocol study that attempted to verify both the efficacy of activity-oriented proprioceptive antiedema therapy (TAPA), as compared to conventional treatments such as decongestive lymphedema therapy (DLT) or complex physical therapy (CPT), as well as its efficiency in terms of cost-effectiveness, for patients affected by breast-cancer-related arm lymphedema. A total of 64 women diagnosed with breast-cancer-related arm lymphedema will participate in the study; the intervention for the experimental group will be the same for stage I or II and will involve neuro-dynamic exercises orientated to the activity, proprioceptive neuromuscular facilitation activities, and proprioceptive anti-edema bandaging. The control group’s intervention will vary based on the stage; stage I will include preventive measures, skincare, and prescribed exercise training at the lymphedema workshop along with compression garments; stage II will include conservative complex decongestive therapy treatment, which includes skin care, multi-layer bandaging, manual lymphatic drainage, and massage therapy. The experimental TAPA treatment seeks to enhance comfort compared to standard treatment by replacing compressive elements with proprioceptive ones. This substitution aims to enable individuals with lymphedema to engage more comfortably in everyday activities. Anticipating the study’s outcomes, TAPA has the potential to establish the first lymphedema rehabilitation protocol for the upper limb secondary to breast cancer, potentially leading to reduced intervention costs [62].

A 2020 study tested two lymphedema prevention interventions and their impact on health-related quality of life (HRQL) in women newly diagnosed with breast cancer (stage I–III). Patients were recruited before breast surgery, and they were randomized into two groups: lymphedema prevention education only (EO) or EO with exercise and physical therapy (LEAP). HRQL was assessed using the Functional Assessment of Cancer Therapy–Breast plus four lymphedema items (FACT-B+4). This 42-item scale consists of six subscales: physical, emotional, functional, and social well-being, other concerns related to breast cancer, and lymphedema symptoms (4 items). A total of 554 patients were enrolled and 547 completed HRQL assessments. The evaluated interventions showed no significant differences in preventing lymphedema or in HRQL outcomes. Women who underwent both axillary node dissection and sentinel node biopsy had higher HRQL compared to those who underwent only one of these procedures. African American women reported more severe lymphedema symptoms but experienced better emotional functioning compared to women from other racial/ethnic groups [17].

In 2022, a study was conducted by M. Hemmati et al. on the effect of combined complex decongestive therapy (CDT) with electrotherapy modalities including ultrasound and faradic currents in patients suffering from breast-cancer-related lymphedema (BCRL). The participants were randomly assigned to three treatment groups: the control group (receiving CDT therapy), the ultrasound group (receiving CDT therapy and therapeutic ultrasound), and the faradic group (receiving CDT therapy and faradic current). The authors conducted a pilot trial with 39 patients, each group consisting of 13 subjects. All participants in the three groups underwent a total of 10 treatment sessions, with 5 sessions conducted per week. The treatment protocol for each group involved a standard CDT, which included manual lymph drainage (MLD), compression therapy using a short stretch bandage, skincare, and lymphedema exercises. The CDT procedure was performed for 1 h per day. In the ultrasound group, patients underwent treatment involving CDT and 1 MHz, 2 W/cm^2^ pulsed ultrasound. The ultrasound application targeted specific points: the midpoint between the elbow joint and the acromion, the biceps lateral tendon in the elbow joint, the midpoint between the olecranon and ulnar styloid (on both the anterior and posterior surfaces of the forearm), and the anterior part of the wrist, each area for 3 min. In the faradic group, patients underwent CDT and faradic current using a stimulator. The faradism under pressure was administered at a frequency of 30 Hz, a duration of 300 µs, an interval of 2 s, and a time of 5 s on both the flexor and extensor forearm muscles of the affected upper extremity, with each surface receiving treatment for 10 min. The treatment session duration for the control group was 1 h. In contrast, the ultrasound group had a session length of about 1 h and 15 min, and the faradic group had a session length of about 1 h and 20 min. After the treatment, improvement was observed in lymphedema volume, pain, and functional disability across all three groups, and there was a significant difference between the groups (*p* < 0.05). Changes in limb circumference after the treatment were not significantly different among the three groups. The combination of CDT with electrotherapy modalities, faradic current, or ultrasound can lead to a more substantial reduction in lymphedema volume, pain, and functional disability in patients with BCRL [11].

Another study conducted in 2023 aimed to compare the impact of myofascial release (MFR) on upper extremity volume in patients with breast-cancer-associated lymphedema (BCRL). The study recruited thirty BCRL patients from outpatient settings in local hospitals in Incheon, Korea. This study was randomized, single-blinded (participants), and patients were divided into two groups. Patients in Group A underwent MFR with complex decongestive therapy (CDT) for 4 weeks, followed by a 4-week washout period, after which they received placebo MFR with CDT for another 4 weeks. Group B received the interventions in the opposite order. Both groups participated in 60-min sessions, occurring twice a week. These sessions consisted of either MFR or placebo MFR for the initial 30 min followed by CDT for the remaining 30 min. To evaluate intervention effectiveness, measurements were taken at four points: week 0 before treatment (T0), week 4 after the first treatment (T1), week 8 after the washout period (T2), and week 12 after the second treatment. The primary outcome was upper limb volume, with subjective pain, shoulder range of motion (ROM), chest mobility, shoulder function, and quality of life (QoL) as secondary outcomes. They were assessed before and at the end of each intervention period. Results suggest that MFR-based treatment may yield a positive effect on upper limb volume, pain, shoulder ROM, shoulder function, and QoL in BCRL patients. There was a clinically significant improvement in shoulder function, attributed to reduced edema volume and pain, improved ROM, and chest mobility. MFR-based treatment is acknowledged as a significant component in BCRL rehabilitation. Furthermore, MFR-based treatment appears to be safe for the studied patients [9].

In 2023, Y. F. Sui realized a case report of a breast cancer survivor who had experienced persistent edema in the left upper limb for more than 15 years. The patient was successfully treated by a combination of conventional rehabilitation (utilizing a seven-step decongestion therapy) and a complete rehabilitation program, which involved the seven-step decongestion therapy, core and respiratory function training, and functional brace wearing. The effectiveness of the rehabilitation therapy was evaluated through a comprehensive process. Despite undergoing the conventional rehabilitation program for one month, the patient experienced only limited improvement. Following an additional month of comprehensive rehabilitation treatment, significant improvements were observed in both lymphedema and the overall function of the left upper limb. The patient’s progress was assessed by measuring a notable reduction in arm circumference. Improvements in joint flexibility were noted and manual muscular strength tests indicated an increase in strength from grade 4 to grade 5. The patient’s quality of life was substantially improved, as demonstrated by the increase in the Activities of Daily Living score from 95 to 100 points, an increase in the Functional Assessment of Cancer Therapy: Breast score from 53 to 79 points, and a decrease in the Kessler Psychological Distress Scale score from 24 to 17 points. Although seven-step decongestion therapy has demonstrated effectiveness in reducing upper-limb lymphedema resulting from breast cancer surgery, its efficacy may be limited when addressing more chronic cases of the condition. Nevertheless, when integrated with core and respiratory function training and functional brace-wearing, seven-step decongestion therapy has proven to be even more effective in reducing lymphedema and also enhances limb function, ultimately resulting in substantial improvements in the individual’s quality of life [63].

A 2023 systematic review quantified the effects of conservative rehabilitation interventions on health-related quality of life in women with upper limb lymphedema secondary to breast cancer. Breast-cancer-related lymphedema (BCRL) affecting the upper limb is a prevalent condition among women undergoing breast cancer treatment, leading to significant alterations in patients’ daily lives and a decline in their health-related quality of life (HRQoL). This review aimed to determine the impact, based on current available evidence, of various conservative interventions in the rehabilitation of BCRL in the upper limb in women. The review suggests that the most recommended approach for the improvement of HRQoL involves a complex decongestive technique without manual lymphatic drainage. Despite the existence of clinical trials demonstrating the effectiveness of various treatments, the results of the positive effects on HRQoL remain a controversial subject. Some of the selected studies indicated a greater short-term enhancement in the physical, functional, and general aspects of the HRQoL among patients who had undergone early home physiotherapy, psychosocial support, or exercise. However, it is essential to continue the development of studies and clinical trials that can offer insights and guidance for therapeutic decisions aimed at enhancing the health-related quality of life (HRQoL) in women affected by BCRL in the upper limbs [64].

De Vrieze et al. analyzed whether manual lymphatic drainage with or without fluoroscopic guidance improves the effect of decongestive lymphatic therapy (DLT) in people with breast-cancer-related lymphedema. A total of 194 patients with unilateral chronic BCRL participated in the study. All participants received standard DLT including education, skin care, compression therapy, and exercises. Participants were randomly assigned to also receive fluoroscopy-guided manual lymphatic drainage (MLD) (n = 65), traditional MLD (n = 64), or placebo MLD (n = 65); during the 3-week intensive phase, participants underwent 14 physiotherapy sessions, followed by 17 sessions during the 6-month maintenance phase. Both traditional MLD and fluoroscopy-guided MLD, when used as adjuncts to complete DLT, did not demonstrate superiority over placebo MLD in decreasing arm/hand volume or fluid accumulation at the shoulder/trunk level in the studied patients [10].

### 3.5. Balneotherapy and Cancer

Balneotherapy uses, in addition to the physical–mechanical effects of water, its chemical effects, which are different depending on the mineral salts contained. The effects of hydrotherapy are dependent on water temperature, while physical exercise in water has the role of improving stretching, muscle strength and range of motion. Thus, hydrostatic pressure and buoyancy are used to improve the symptoms of patients with musculoskeletal, orthopedic or neurological conditions [65]. The data regarding the effectiveness of these therapies in the case of oncological patients are quite limited, with the discussions balancing the risk/benefit ratio; on the one hand, therapy can be effective in relieving pain and fatigue as well as in improving mobility; on the other hand, it can worsen lymphedema and increase the risk of infections [66]. In the case of patients with breast cancer, balneotherapy, which included warm wraps with mud and baths in carbonated mineral water, reduced fatigue and improved the patients’ quality of life. Hyperthermia did not negatively influence the evolution of tumor marker CA 15-3 [67].

Reger et al. conducted a systematic review, published in 2022, of 430 cancer patients aged 18–78 composed of 378 women and 52 men. Patients with breast cancer (n = 206, 4 studies) and gynecological cancer (n = 113, 3 studies) predominated; patients with colorectal cancer (n = 40, 1 study) and a mixed group of patients with various cancers (bladder, prostate, lung, liver) (n = 38, 2 studies) were also included [68]. The most frequently used intervention was represented by aquatic exercises such as aquatic lymphatic therapy, including aerobic exercises, mobilizations and stretching in pools of water, partial foot baths or general baths. The duration of the spa treatments was between 8 weeks and 3 months. In almost all studies, spa therapy was used as a complementary therapy to relieve pain or morbidity associated with the disease or therapeutic intervention (surgical treatments, chemotherapy or radiotherapy), and in two of the studies as a palliative method to relieve the symptoms of incurable cancer. The patients were evaluated from the point of view of joint mobilization, well-being and quality of life, the presence or absence of lymphedema, painful trigger points, body image and the patients’ involvement in socio-professional life. The patients were also evaluated in terms of vital parameters: temperature, heart rate, blood pressure and the presence of anxiety. The types of treatment, their frequency and duration, and the possible adverse reactions associated with the therapeutic act were analyzed; the patients were characterized according to the type and stage of cancer, the type of treatment (radio- and chemotherapy, hormonal treatment, surgical treatment), age, sex and other demographic data. The conclusion of this systematic review was that no clear recommendations can be made regarding the effectiveness of balneotherapy and hydrotherapy in the case of oncological patients. There is a moderate level of evidence indicating ways to improve quality of life and lymphedema in patients with various cancers. The beneficial effects were highlighted for active training in water. not for passive mobilizations. Future studies are needed regarding the safety of using mineral waters with high temperatures [68].

### 3.6. Laser Therapy and Electrotherapy in Patients with Cancer

Low-level laser therapy (LLLT) or photobiomodulation therapy (PBMT) is a type of phototherapy that uses less than 500 mW of power. Because it has biostimulation effects, it is also called photobiomiodulation therapy, and, unlike high-level laser therapy, it does not cause tissue heating. Aside from its biostimulatory effects, it has been documented that LLLT has anti-inflammatory effects [69,70,71,72] and stimulates blood flow by releasing nitric oxide from nitrosyl complexes [73]. Therefore, given its beneficial biological effects, LLLT can be a good alternative for the treatment of chemoradiotherapy-related symptoms and disorders in cancer patients.

One of the main complications of breast cancer treatment is lymphedema, affecting up to 40% of patients (6). The occurrence of lymphedema can happen either during treatment or after the treatment is completed [74]. A randomized clinical trial [44] including 46 breast cancer survivors with treatment-related lymphedema compared the effectiveness of three therapeutic combinations: LLLT alone (20 min/session), LLLT in combination with manual lymphatic drainage (MLD) (20 + 20 min/session) and MLD alone (40 min/session). Compression bandaging was applied after each treatment regardless of the therapeutic method priorly used. Three main outcomes were looked for: arm volume, symptoms and quality of life. Arm size was assessed using bioelectrical impedance (L-Dex) for extracellular fluid and circumferential measurement for arm volume. No significant difference was observed among the groups (*p* = 0.984 for L-Dex values, *p* = 0.422 for circumferential measurement), but in terms of both L-Dex values and circumferential measurement, there was a statistically significant reduction in all of the groups from their baseline values. Physical and psychological symptoms were assessed using the Lymphedema Symptom Intensity and Distress Scale—Arm (LSIDS-A) checklist. Similar to the findings for arm volume, changes in symptom number, type or burden did not occur with statistically significant differences among the groups, while the symptom burden was statistically significantly lower for all groups from baseline values. Simultaneously, the participants filled in a skin assessment checklist, the number of conditions reported being typically quite low. However, a statistically significant greater reduction in that number was observed within LLLT and combined LLLT-MLD groups than within the MLD group. In terms of quality of life (QOL), little change occurred over the course of the study because QOL and indicators of functioning were relatively high for all participants at baseline entry, and no statistically significant differences were found among the groups. After comparing three main outcomes (arm volume, symptoms and quality of life), the research team found no significant difference between the groups, while underlining that there was a beneficial outcome in all groups from baseline values. Given the fact that LLLT therapy has shorter treatment times (20 min as opposed to 40 min in MLD or LLLT-MLD combined), it could represent an alternative for treating more patients on a daily basis, especially in centers where the waiting list is lengthy. Because compression bandaging was used in all three groups, the authors note that the effects observed can be attributed to this intervention alone, and if this is true, it can contribute to a reduction in patient burden and duration of treatment sessions.

Legouté et al. [22] tested the effectiveness of LLLT for the prevention and treatment of chemoradiotherapy-induced mucositis in head and neck cancer. The patients were treated daily following a radiotherapy session using the curative dose recommended by the Multinational Association of Supportive Care In Cancer (MASCC) of 4 J/cm^2^. They found no statistically significant difference between the LLLT/PBMT and placebo arm neither in preventing nor in the final outcome of oral mucositis (OM). Alongside oral mucositis, which was the primary endpoint, the authors also evaluated secondary endpoints, including nutritional status, pain, quality of life, chemo-radiotherapy (CRT) compliance, laser therapy compliance and tolerance, finding no statistically significant difference between the two groups. The research team concluded that while LLLT/PBMT was well tolerated with a good safety profile, there are also data from the literature that emphasize the potential risk of enhancing the malignant potential of primary tumor [75]; thus, there is a need for other large studies to investigate the risk/benefit ratio of LLLT in cancer patients.

LLLT/PBMT applications and treatment protocols in the management of side effects of CRT in head and neck cancer were proposed by Zecha et al. [76]. The team’s review of the literature found that LLLT/PBMT was effective for reducing OM prevalence, duration, severity and its associated pain, finally proposing an energy density of no more than 6 J/cm^2^, setting this upper limit as a precaution since they found no clinical data defining a safe upper limit. They also noted that LLLT/PBMT can be administered extraorally when there is OM of the buccal mucosa, vestibule, and inner epithelial surfaces of the lips [77]. Because of its ability to reduce inflammation and pain associated with OM, along with its ability to control exuberant fibrosis [78], LLLT/PBMT may also have a beneficial role in the management of head-and-neck-cancer-associated dysphagia. Testing the effectiveness of radiotherapy-induced dermatitis, the authors found contradictory results but noted that LLLT/PBMT may be a useful tool in reducing the prevalence and/or severity of radiation therapy [79,80,81], given the fact that laser therapy has an anti-inflammatory effect [69,70,71,72] and promotes wound healing [73,82]. Regarding hyposalivation and xerostomia after radiotherapy, the research team concluded that LLLT/PBMT may be beneficial for the prevention of hyposalivation and/or xerostomia [83], as long as there is no irreversible acinar atrophy and/or fibrosis of the salivary glands [84]. Furthermore, given its anti-inflammatory and salivary flow stimulating effects, patients with voice and speech alterations in head and neck cancer patients could benefit from LLLT/PBMT. LLLT/PBMT was found to be effective in reducing lymphedema in patients following breast cancer treatment [85,86,87]; therefore, LLLT/PBMT was proposed as a potential treatment alternative for lymphedema associated with head and neck cancer [88]. Despite the fact that the research team was unable to find clinical studies on the effects of LLLT/PBMT on radiotherapy-induced jaw osteonecrosis, it was highlighted that there are data indicating the beneficial role of LLLT/PBMT in both radiotherapy-related bone damage [89,90] and medication-related osteonecrosis of the jaw [89,90,91,92,93,94], thus necessitating specifically exploring the role of LLLT/PBMT in radiotherapy-induced jaw osteonecrosis. The authors did not find any report of LLLT/PBMT for radiotherapy-induced trismus but noted that there are studies reporting a reduction in muscle spasms after oral surgery when LLLT/PBMT was used [95,96]. Regarding dysgeusia in cancer patients, no published studies on the effect of LLLT/PBMT were found; however, it was noted that taste alterations as seen in neurologically mediated burning mouth syndrome were ameliorated after LLLT/PBMT administration [97].

Transcutaneous electrical nerve stimulation (TENS) is a type of electrotherapy that uses low-frequency current (<1000 Hz) through two electrodes placed on the skin that stimulate the nerves. TENS devices have the advantages of being inexpensive, user-friendly (there is no need for a therapist), and small in size. There is also little risk of overdose. There are a few contraindications for the usage of TENS; it should not be applied on areas where skin lesions or allodynia are present [98].

Chemotherapy-induced peripheral neuropathy (CIPN) is one of the side effects of cancer treatment agents, including platins, taxans and vinca alkaloids [99,100]. One of the proposed pathways by which CIPN produces pain is by enhancing excitability and impairing inhibition in the central nervous system [101,102,103,104], making interventions that reduce central excitability and enhance central inhibition a good alternative for the improvement of CIPN symptoms, one of them being TENS.

Gewandter et al. [105] wanted to assess the feasibility of wireless TENS devices for CIPN. The European Organisation for Research and Treatment for Cancer-CIPN20 (EORTC-CIPN20), The Short Form McGill Pain Questionnaire-2 (SF-MPQ-2), numeric rating scale (NRS) and The Utah Early Neuropathy Score (UENS) were used to measure the final outcomes. There were statistically significant differences in both EORTC-CIPN20 (*p* = 0.04) and SF-MPQ-2 (*p* = 0.002) scores. Additionally, NRS daily scores for pain, tingling, numbness and cramping improved statistically significantly. Neuropathy signs assessed by the UENS score did not improve statistically significantly from baseline after 6 weeks of treatment with TENS. As for the adverse effects, the research team noted a few mild side effects that could have been attributed to TENS (contact dermatitis, worsening or new paresthesias and pain or cramping in the lower limbs and tightness in the lower limbs). All mild adverse effects resolved after TENS treatment was discontinued or the duration was lowered; thus, the likelihood of TENS permanently harming CIPN patients is low. Another significant observation made by the authors was that although improvement in numbness was smaller (i.e., 20%), there is a lack of pharmacological treatments for numbness, so TENS could be especially beneficial in this particular situation.

Püsküllüoğlu et al. [106] reviewed the literature to assess the effectiveness of TENS on CIPN in cancer patients. Their study included seven trials, all indicating, with a statistically significant difference, that TENS may show effectiveness (at a certain degree) in pain treatment in the CIPN patient subpopulation. Moreover, two of the studies showed that analgesics consumption (opioid and non-opioids) was statistically significantly lower in the TENS group compared to placebo. Similarly to the study conducted by Gewandter et al., TENS was considered to be a safe and easy-to-use procedure.

TENS improves the symptoms of polyneuropathy secondary to chemotherapy, in association or not with other therapeutic modalities, such as home-based high-tone external muscle stimulation therapy (HTEMS), the results being visible in changes in sensitivity to vibrations and temperature, changes in osteo-tendinous reflexes, the perception of touch, muscle strength and improving the quality of life of oncological patients [107].

In addition to the beneficial effects gained from improving symptoms of polyneuropathy secondary to cancer treatments [106,107], TENS is an effective treatment for lower urinary tract symptoms secondary to radical hysterectomy and pelvic lymphadenectomy in patients diagnosed with early cervical cancer. The patients were characterized according to their menopausal status and the modality of the surgical intervention; they were randomized into two groups and were followed up to 24 months after the surgical intervention. The patients were evaluated regarding the rate of improvement of urinary function (by measuring residual urine), urodynamic parameters, urinary incontinence, pelvic function and quality of life [108].

### 3.7. Therapeutic Ultrasound for Patients with Cancer

Therapeutic ultrasound (US) is used in physical therapy for pain relief and tissue repair. It can be delivered either continuously or pulsed with various implications on the tissue level. Continuous US is absorbed and heats the tissues, resulting in vasodilation, increased metabolism and analgesia. Pulsed US modulates cell membrane permeability and promotes tissue regeneration [109,110]. Moreover, ultrasound mechanical waves produce molecule vibration and have been shown to accelerate the healing of fractures, ligaments and cartilage as well as axonal regrowth and reinnervation and nerve conduction after nerve injury [111].

Low-intensity ultrasound (LIUS) was studied by Elgohary et al. in the treatment of trismus and temporo-mandibular joint pain in head and neck cancer survivors. The authors found that LIUS combined with therapeutic exercise had superior effects on pain, quality of life and maximum mouth opening in comparison with low-level laser therapy combined with therapeutic exercise or therapeutic exercise alone [27].

Oanzi et al. studied the effects of therapeutic ultrasound on oxaliplatin-induced CINP and sensory disturbance in the hands and feet of colorectal cancer patients. The application of LIUS for 5 min every day for 10 days was evaluated through pain questionnaires that showed significant improvement at the end of the intervention but not after 6 weeks. There was no difference regarding the quality of life, protective sensation and temperature [112].

### 3.8. Oxygen Therapy Using Specific Training of Hyperoxia/Hypoxia in Cancer Patients

In 2017, M. Schumann et al. set out to investigate the feasibility, safety, and tolerance of high-intensity interval training (HIIT) in cancer patients undergoing chemotherapy, comparing its effects in hyperoxia to those in intermittent hyperoxia–hypoxia. A total of forty-eight cancer patients were randomly assigned to one of three intervention groups or a control group with no training. Participants in the intervention groups performed HIIT on a cycle ergometer, experiencing either hyperoxia, intermittent hyperoxia hypoxia, or normoxia. The prescribed training exercise involved four weeks of HIIT on a bike ergometer. The groups were divided as follows: normoxia: HIIT conducted in normoxia (FiO^2^ 0.21); hyperoxia: HIIT with both exercise and rest periods carried out in hyperoxia (FiO^2^ 0.3); hyperoxia/hypoxia: HIIT with exercise performed in hyperoxia (FiO^2^ 0.3) and rest periods in hypoxia (FiO^2^ 0.15); control group: no prescribed training and no alterations in inspired oxygen concentrations. The FiO^2^ levels were concealed from the participants, and individuals in the normoxia group underwent the training without wearing a mask. The training sessions were performed twice a week and comprised 4–5 sets of 2-min high-intensity training intervals at 80% of the Wmax (maximal Wattage), with each set followed by a 3-min recovery period at 40% of Wmax. Feasibility was evaluated through trial completion, training compliance and adherence, program tolerance, and patient safety. It is reasonable to assume that this approach could enhance patients’ adherence to regular exercise, while it may provide beneficial physiological adaptations [113]. Additionally, a review conducted by Uzun et al. highlights the advantages of hypoxic–hyperoxic and hypoxic–normoxic therapy across various conditions and populations. Protocols involving exposure to mild hypoxia in short episodes, when administered over several days or weeks, can yield significant beneficial outcomes. Hypoxia–hyperoxia and hypoxia–normoxia appear to be promising non-pharmacological intervention approaches that are well tolerated by patients and can be included in the therapeutic management of various medical conditions [114].

## 4. Discussion

Medical rehabilitation is based on a detailed assessment of the oncology patient’s needs and health. The rehabilitation program is personalized and established according to the type of cancer, the stage of the disease, the patient’s tolerance and the associated pathologies. Regular physical exercise is essential for the rehabilitation of cancer patients. It can help improve overall health, physical strength, cardiac function, respiratory function and mobility. After mastectomy, rehabilitation may include exercises to restore arm and shoulder mobility. For patients who have undergone radiotherapy or chemotherapy, rehabilitation may involve managing fatigue and other treatment-related side effects. Rehabilitation after prostatectomy may include exercises to improve erectile function and bladder control. For patients with advanced prostate cancer, rehabilitation may include managing pain and other associated symptoms. After colorectal surgery, rehabilitation may include exercises to restore bowel function and anal continence. Respiratory rehabilitation is essential for lung cancer patients, including breathing exercises and improvement of lung capacity. For those undergoing lung cancer surgery, rehabilitation may also include exercises to reduce the risk of postoperative pulmonary complications. Rehabilitation for pancreatic cancer may involve managing pain and abdominal discomfort associated with the disease and its treatment [21,28,29,38].

Patient education about their disease, treatment, and the importance of rehabilitation can enhance their understanding and acceptance of this aspect of their care. Informed patients are more likely to be compliant with rehabilitation treatment. Medical rehabilitation techniques are non-invasive and accepted by patients, with a high level of compliance because they are not painful and do not cause discomfort. Compliance of oncology patients with medical rehabilitation can be influenced by a variety of factors unique to this population and their experience with the disease and treatment. Informing patients about the benefits of medical rehabilitation in terms of improving quality of life, reducing symptoms, and increasing functionality can increase the likelihood of compliance with the rehabilitation program. Open and empathetic communication between the patient and the medical team can contribute to the understanding and acceptance of the rehabilitation process. Efficient management of treatment side effects can improve patient compliance. Managing pain, fatigue, or other symptoms can make the rehabilitation process more accessible and tolerable for the patient. Addressing the patient’s emotional needs, such as anxiety, depression, or fear, can contribute to increased compliance. Counseling and emotional support can help the patient better cope with challenges and remain motivated during the rehabilitation process [15,16,20,41,64].

There is a wide variety of individual needs and circumstances among oncology patients, including cancer type and stage, age, general health status and other comorbidities. A detailed exploration of the specific implementation of various rehabilitation methods can help tailor treatment programs to better meet the unique needs of each patient. It is important to thoroughly explore the applicability of different rehabilitation methods to address side effects and improve the recovery of these patients. The specific implementation of rehabilitation methods must be adapted to each stage of treatment and the specific needs of patients in each phase. Clinical studies and qualitative research can provide valuable insights into the applicability of rehabilitation methods in certain groups of oncology patients. These ideas emphasize the importance of a detailed exploration of the specific implementation of medical rehabilitation methods and their applicability to different oncology patient groups to ensure optimal care and improve patient outcomes [2,46,65]. We believe that further studies are necessary to explore, in more detail, the implementation and application of rehabilitation methods in certain patient groups. It is necessary to form a multidisciplinary team between the physical medicine and rehabilitation physician and the oncologist, in the context of oncology patients requiring medical rehabilitation.

Exercise as a beneficial intervention is clearly demonstrated in many papers. Several studies highlight the positive impact of exercise interventions on cancer patients’ well-being. Many authors demonstrated that moderate-to-vigorous aerobic and resistance exercises significantly improved quality of life, reduced depression, decreased fatigue, and enhanced physical fitness in breast cancer survivors and patients undergoing chemo-radiotherapy [20,21,25]. It was also shown that endurance and balance training exercises can reduce sensory symptoms and improve the functional status in patients with chemotherapy-induced peripheral neuropathy [24]. These findings collectively emphasize the role of exercise in mitigating cancer-related symptoms and improving overall quality of life. Resistance training appears to be a promising approach for cancer patients, as some studies have highlighted [21,41]. These studies found that resistance training led to significant reductions in fatigue and improvements in quality of life for breast cancer patients receiving adjuvant radiotherapy and individuals dealing with pancreatic cancer. Resistance training offers an alternative exercise modality that can positively impact cancer-related symptoms. Regarding multimodal exercise programs, in a study from 2021 [15], it was demonstrated that a combination of aerobic, resistance, and flexibility exercises improved physical fitness and health-related quality of life in head and neck cancer patients undergoing chemotherapy. This suggests that multimodal exercise programs that incorporate various exercise components may yield comprehensive benefits for cancer patients.

Regarding aquatic therapy, despite the similar intensity, frequency, and duration for the land- and water-based programs during the exercise period [46], it was found that aquatic physical therapy, consisting of various exercise modes—particularly endurance and strength exercises—is more effective than land-based exercise for improving quality of life associated with breast cancer treatment. It has also been found that employing a water exercise intervention is more successful in enhancing mental health and lowering side effects related to breast cancer therapy in contrast to yoga and Pilates therapies [23]. This could be because working out with other women who went through similar things led to the formation of new friendships. Additionally, the exercise sessions’ intriguing anecdotes, warm water, and relaxing music might have elevated participants’ moods during the intervention period. Aquatic therapy stands out as a promising intervention by reducing fatigue compared to standard care and improving quality of life compared to land-based training in cancer patients. This indicates that the unique properties of water-based exercise may offer advantages in managing cancer-related symptoms.

Physical medicine still has many uncertainties related to the methodology of applying electric currents, regarding the indications, contraindications and therapeutic precautions in the case of oncological patients. Electrotherapy is contraindicated due to the lack of clear scientific evidence in favor of the application of electric currents [115]. Electrotherapy should not be used in the area of the tumor (with the exception of palliative care) in the case of bleeding tissues or hemorrhages or in areas at risk of pathological fractures. Electrotherapy can be applied with caution in the case of areas with sensitivity or circulatory disorders and in the case of devitalized tissues after recent radiotherapy or acute dermatological lesions (eczema, dermatitis) [116]. Zheng et al. draw attention to the safety and precautions of rehabilitation treatments in the case of cancer patients, emphasizing the possibility of multiorgan compromise and the side effects of radiotherapy [117].

There are two different aspects from the point of view of applying treatments to oncological patients; the first refers to the application of some treatment methods in the case of patients diagnosed with various cancers who present pain or a limitation in function, and the second aspect refers to the treatment of a musculoskeletal condition that occurred as a complication of the disease or oncological treatments (lymphedema, neuropathies, adherents) [118].

The literature highlights some beneficial effects of the use of electric currents in oncology patients, these being represented by the limitation of nerve compression, the relief of pain and lymphedema, the improvement of joint mobility and tissue perfusion, the prevention of dysfunctions, and the reduction in risk factors and complications. Of course, the individual assessment of each patient is important; after excluding the risk of relapse and metastasis, many physiotherapy procedures can be applied safely [119].

Rehabilitation in cancer aims to improve the functional status of patients by participating in social activities and improving their quality of life and includes the application of physical modalities such as electrotherapy and derivative therapies (mechanotherapy, phototherapy), balneotherapy and spa therapy, physical therapy, occupational therapy and aquatic therapy [120,121].

Crevenna et al. published a study on the safety and efficiency of using radial or focused shockwave therapy, in the case of oncological patients, when the malignant tumor is not in the treatment area. These indications are recommended by the International Society for Medical Shockwave Treatment (ISMST) for “common empirically tested clinical use” and as exceptional indications/expert indications [122].

Additionally, along with conventional rehabilitation therapy, telerehabilitation, used more in the case of neurological patients and during pandemic periods [120], has proven its applicability and effectiveness in the case of cancer patients, representing an easy therapy method used to improve the functional status of patients [123,124].

Balneo- and hydrotherapy are alternative methods of treatment in the case of oncological patients, regarding the improvement of some symptoms and adverse effects of cancer therapies, such as the improvement of pain, fatigue, lymphedema and limitation of physical function [67]. Active forms of aquatic physiotherapy are indicated in these cases. Before starting the therapy, it is recommended for the oncologist to evaluate the patient and assess the risks and contraindications [68].

Cancer-related fatigue (CRF) is a prevalent and debilitating symptom experienced by cancer survivors, which can be attributed to various causes such as the cancer itself, chemotherapy, comorbidities, nutritional state, and functional status. This type of fatigue cannot be relieved by rest as it worsens with a decrease in physical activity, creating a vicious cycle [12,26,33,49].

American ginseng and fermented Korean red ginseng showed similar positive effects on CRF compared to placebo, the effects being persistent after 2 months of intake. Additionally, two types of massage therapy, Japanese and Swedish, were found to relieve CRF, compared to no intervention or other types of massage. Moreover, ginger was found to lessen fatigue better compared to placebo in a study evaluating the symptoms in the first and second course of chemotherapy in 51 patients [53]. Some studies have demonstrated the effectiveness of massage therapy in alleviating cancer-related symptoms such as pain, fatigue, anxiety and chemotherapy-induced peripheral neuropathy, while others show no differences in effects on depression, mood disturbance, psychological distress, nausea, fatigue, physical symptom distress, or quality of life when compared with no massage, as studies are limited and lack high-quality evidence [49]. It was also found that massage therapy reduced pain, particularly during ambulation, in children with cancer [47]. It was demonstrated that massage therapy was effective in treating pain, fatigue, and anxiety in cancer patients [48]. Anma massage therapy [26] and Swedish massage therapy [33] were also found to provide relief for cancer-related fatigue, and another study [12] explored different massage frequencies, suggesting that tailored massage schedules can optimize results for chemotherapy-induced peripheral neuropathy. While the evidence supports massage therapy’s role in symptom management, a review [49] highlights the need for further research to confirm its benefits.

Additionally, aquatic therapy should be considered a better option for gaining greater aerobic fitness in cancer patients dealing with CRF, since a training effect may be obtained at a much slower speed than on land, as the water’s buoyant force eases the strain on the weight-bearing joints and permits upper limb joint mobility [16].

In the months that follow adjuvant chemotherapy and radiation therapy, cancer patients are more likely to experience certain cancer-related symptoms or symptoms related to specific cancer treatments, such as nausea, chemotherapy-induced peripheral neuropathy, depression, anxiety, sleep issues, sexual dysfunction, etc. These side effects can lower QoL and have a detrimental effect on social and domestic activities [12,35].

Medicinal plants and phytochemicals, such as ginger, ginseng, and others, contain bioactive compounds that may offer benefits to cancer patients. Ginger has shown promise in reducing nausea and fatigue [38,40,53], while ginseng has been associated with reduced cancer-related fatigue [2,49]. The use of curcumin in combination with chemotherapy appears to be safe and may improve fatigue [28]. Specific compounds found in plants, such as rosmarinic acid (RA), carotenoids, and phosphoinositol hexaphosphate (IP6), have demonstrated anti-tumor properties and potential in cancer prevention. However, further research is needed to establish their efficacy in clinical settings [59].

Different items in QoL assessment were found to be improved by aquatic therapy and massage therapy, as well as fermented Korean red ginseng or ginger. When comparing the treatment group to the chemotherapy-alone group, a significant improvement in cancer-related symptoms, psychological status, physical conditions, quality of life, and adverse effects caused by chemotherapy was observed. Moreover, ginger, a traditional remedy used by many cultures for gastrointestinal issues, showed comparable effectiveness to metoclopramide in alleviating chemotherapy-induced nausea and vomiting (CINV) [31,53]. Studies on nanotechnologies from herbal supplements such as echium oil and saw palmetto suggest their potential impact on quality of life and symptom management during cancer treatment. These supplements may serve as complementary approaches to enhance patients’ well-being [34,43].

Traditional Chinese herbal medicine (TCM) has been investigated for its potential benefits in cancer care, examining patients with advanced non-small-cell lung cancer, and improvements in quality-of-life domains (physical and emotional well-being, functional well-being) were found. This suggests that TCM could be a valuable adjunctive therapy for cancer patients compared to best-supporting care as an only treatment [38].

The evidence supporting the use of ginseng, ginger, massage, Chinese herbal medicine (CHM) and aquatic therapy in cancer care is growing, with studies highlighting its positive impact on patients’ physical and emotional well-being, as it can complement conventional care and contribute to an increase in quality of life for cancer patients and survivors.

Regarding these different symptoms that can be found in patients diagnosed with different types of cancers, or with complications after oncological procedures, we identified the potential intervention from diverse nonpharmacological treatments:

### 4.1. Effects on Quality of Life, Fatigue, and Physical Fitness

Dieli-Conwright et al. [25] found that moderate to vigorous aerobic and resistance exercises led to significant improvements in quality of life, reduction in depression, decreased fatigue, and enhanced physical fitness in breast cancer survivors. Samuel et al. [20] reported improved functioning, enhanced quality of life, and reduced levels of fatigue among head and neck cancer patients who received a structured exercise intervention. Resistance exercises during chemotherapy, as shown by Schmidt et al. [42], led to significant benefits in reducing fatigue and enhancing quality of life in breast cancer patients. Steindorf et al. [21] demonstrated the potential of resistance training to alleviate symptoms, enhance physical function, and improve the overall quality of life for pancreatic cancer patients. Endurance and balance training exercises reduced sensory symptoms and improved functional status in chemotherapy-induced peripheral neuropathy patients, as shown by Kneis et al. [24]. Lin et al. [15] found that a combination of aerobic, resistance, and flexibility exercises improved physical fitness and health-related quality of life in head and neck cancer patients undergoing chemotherapy. The UMBRELLA Fit study [14] showed minor improvements in fatigue levels but did not significantly impact the quality of life in breast cancer patients who participated in supervised exercises.

### 4.2. Effects on Reduction in Depression and Anxiety

Dieli-Conwright et al. [25] also reported a reduction in depression among breast cancer survivors who engaged in moderate to vigorous aerobic and resistance exercises. Resistance exercises during chemotherapy were associated with a reduction in depression, as indicated by Schmidt et al. [42].

### 4.3. Effects on Alleviation of Nausea

Hong et al. [18] found that machine-based resistance exercise training reduced symptoms such as nausea and acid reflux in gastrointestinal cancer patients undergoing chemotherapy. Ginger supplementation showed effectiveness in reducing chemotherapy-induced nausea and vomiting, as indicated by a study in 2017 [53].

### 4.4. Effects on Anti-Cancer and Prevention of Cancer-Related Weight Loss

Medicinal plants and phytochemicals, such as rosmarinic acid, carotenoids, and others, have potential anti-cancer effects and may inhibit the progression of tumors [51]. Bovine colostrum supplements have shown potential anti-tumor properties in preclinical studies.

Curcumin supplementation, when combined with chemotherapy, demonstrated safety and potential benefits for patients with metastatic colorectal cancer. IP6 + Ins supplements may enhance the anticancer effects of conventional chemotherapy, control cancer metastases, and improve the quality of life for cancer patients [28].

### 4.5. Effects on Enhancement of Immunity

Ginseng supplementation was associated with enhanced immunity and reduced cancer-related fatigue. Chinese herbal medicine (CHM) showed the potential to improve the quality of life in patients with non-small-cell lung cancer [38]. Some plant-based supplement nanotechnologies, such as phosphorated inositol hexaphosphate, also enhanced immunity and contributed to the destruction of tumor cells (Phosphorylated Inositol Hexaphosphate) [58].

### 4.6. Effects on Androgen Formation

Actaea racemosa (black cohosh) did not stimulate the growth of breast cancer cells but increased androgen formation, potentially alleviating menopausal symptoms. Echium oil supplementation increased androgen levels but did not protect against weight loss induced by radio(chemo)therapy [43].

### 4.7. Effects on Symptom Management

Massage therapy, including Swedish massage therapy and Anma massage therapy, showed effectiveness in reducing pain, fatigue, and anxiety in cancer patients. Saw palmetto (SP) supplementation did not significantly impact lower urinary tract symptoms during radiation therapy for prostate cancer [26,34].

## 5. Conclusions

It is important to emphasize that therapeutic methods should be integrated into a personalized care plan and administered under the supervision and coordination of a medical team. Open communication between the patient and their medical team is essential to tailor these therapies to each individual’s needs and health status.

It is important to emphasize that alternative therapies should not replace conventional medical treatments but rather should complement them. An integrative approach that combines the benefits of traditional treatments with those of alternative therapies can bring the best results for patients. It is also essential that patients always speak with their medical team before starting any form of alternative therapy to ensure proper treatment coordination.

In conclusion, integrative alternative therapies represent a significant component in the rehabilitation of cancer patients, offering a holistic approach that complements conventional treatments. These therapies not only alleviate the adverse symptoms of the treatments but also support the general well-being of patients with a major increase in quality of life. However, it is crucial that we approach these therapies with discernment and integrate them into a well-coordinated medical framework to ensure the best possible care for our patients.

## Figures and Tables

**Table 1:** The keyword combinations used for scoping the international databases.

	PubMed	PEDro	Cochrane	Elsevier	Total
Physical exercises and cancer rehabilitation and quality of life	2787	12	4	5268	8071
Water-based physical exercises and cancer rehabilitation and quality of life	8	0	3	1738	1749
Transcutaneous electrical nerve stimulation and cancer rehabilitation and quality of life	91	0	1	1041	1133
Ultrasound and cancer rehabilitation and quality of life	186	1	26	4818	5031
Laser and cancer rehabilitation and quality of life	43	1	5	3498	3547
Extracorporeal shockwave therapy and cancer rehabilitation and quality of life	15	0	0	485	500
Balneotherapy and cancer rehabilitation and quality of life	1	0	1	70	72
Hypoxia–hyperoxia and cancer rehabilitation and quality of life	1	0	0	97	98
Massage therapy and cancer rehabilitation and quality of life	198	1	0	2215	2414
Cancer quality of life rehabilitation nanotechnology	6	0	1	523	530
Cancer supplements and quality of life	1637	5	1064	119,132	121,838
Lymphedema and cancer rehabilitation and quality of life	468	10	84	1104	1666
**Total**	5441	30	1189	139,989	**146,649**

**Table 2:** The study description.

Authors	Year	Type of Cancer/ Clinical Conditions after Oncological Treatments	Intervention	Surveys	Conclusions
Kim et al. [9]	2023	Breast	Myofascial release	NRS DASH	Myofascial release improved shoulder function, range of motion and chest mobility.
De Vrieze et al. [10]	2022	Breast	Decongestive lymphatic therapy Fluoroscopy-guided manual lymphatic drainage	Self-developed questionnaire	Fluoroscopy-guided manual lymphatic drainage did not show significant benefits in limb lymphedema, in addition to other parts of decongestive lymphatic therapy.
Hemmati et al. [11]	2022	Breast	Complex decongestive therapy Electrotherapy (ultrasound and faradic currents)	NRS DASH	Complex decongestive therapy combined with electrotherapy showed a more significant reduction in lymphedema pain, volume and disability.
Lopez et al. [12]	2022	Peripheral neuropathy	Swedish massage therapy	PQAS	Three or four sessions per week resulted in best outcomes in symptom relief.
Bruce et al. [13]	2021	Breast	Strengthening, physical activity, stretching and behavioral change techniques	DASH questionnaire	Exercises proved to be clinically and cost-effective, reducing upper limb disability in breast cancer patients at risk of postoperative complications.
Gal et al. [14]	2021	Breast	Aerobic exercises, Resistance training	Global QoL EORTC QLQ-C30 MFI-20	Half of the patients accepted the exercise intervention, which resulted in there being no impact on quality of life and only a small benefit regarding fatigue levels.
Lin et al. [15]	2021	Head and neck	Moderate-intensity aerobic, resistance and flexibility exercises	EORTC-QLQ-C30 EORTC-QLQ-H&N35	The intervention showed benefits in physical fitness and health-related quality of life.
Wang et al. [16]	2021	Breast	Flexibility exercises Resistance exercises	FACT-ES	Significant improvements in terms of endurance and strength, but no significant improvements in physical functioning or in quality of life.
Naughton et al. [17]	2021	Breast	Lymphedema prevention education only or lymphedema prevention education with exercises and physical therapy	FACT-B	The interventions showed no difference between them in preventing lymphedema.
Hong et al. [18]	2020	Gastro-intestinal	Resistance training	EORTC-QLQ-C30	The intervention reduced the apparition of nausea and acid reflux, improved physical function and eased fatigue and appetite loss.
Wu et al. [19]	2020	Gynecologic	Rehabilitation exercises Complex decongestive therapy	EORTC QLQ-C30 BFI	Complex decongestive therapy in combination with rehabilitation exercise decreased limb lymphedema and fatigue levels and improved quality of life.
Samuel et al. [20]	2019	Head and neck	Aerobic and dynamic resistance exercises	SF-36 NCCN scale	The intervention had a positive impact on functioning, quality of life and on fatigue levels.
Steindorf et al. [21]	2019	Gastro-intestinal	Supervised progressive resistance training group Home progressive resistance training group	EORTC QLQ-C30 EORTC QLQ-PAN26	Resistance training may be a promising method for symptom relief, improving physical function and quality of life.
Legouté et al. [22]	2019	Head and neck	Low-level laser therapy	EORTC QLQ-H&N35	The therapy proved to be well tolerated and had a good safety profile for oral mucositis.
Odynets et al. [23]	2019	Breast	Water-based exercises Pilates exercises Yoga exercises	FACT-B	Water-based exercises showed more benefits in increasing well-being and decreasing negative symptoms related to breast-cancer-specific treatment.
Kneis et al. [24]	2019	Peripheral neuropathy	Endurance and balance training	EORTC-QLQ-C30 EORTC QLQ-CIPN20	Endurance training led to a reduction in sensory symptoms, and balance training further enhanced patients’ functional status.
Dieli-Conwright et al. [25]	2018	Breast	Aerobic exercises, resistance exercises	SF-36 FACT-B CES-D	Improvements in terms of quality of life, depression, fatigue, and physical fitness.
Donoyama et al. [26]	2018	Gynecologic	Japanese massage therapy	EORTC QLQ-C30 HADS POMS MAC	Improvements in quality of life, fatigue levels and insomnia.
Elgohary et al. [27]	2018	Head and neck	Low-intensity ultrasound Low-level laser therapy Traditional exercise therapy	UW-QOL VAS	Ultrasound and exercise therapy proved to be more effective than laser therapy in pain reduction and trismus.
Howells et al. [28]	2018	Gastro-intestinal	Curcumin (2 g orally)	EORTC-QLQ C-30	Curcumin proved to be safe and tolerable in metastatic colorectal cancer patients undergoing chemotherapy.
Hojan et al. [29]	2017	Prostate	Strength training Treadmills Cycle ergometers	FACT-F EORTC QLQ-C30 EORTC QLQ-PR25	Physical activity ameliorates functional capacity, reduces inflammatory biomarkers and fatigue, and also has a beneficial effect on quality of life.
Mijwel et al. [30]	2017	Breast	High-intensity interval resistance training; moderate and high-intensity aerobic interval training	Piper Fatigue Scale EORTC-QLQ-C30 The Memorial Symptom Assessment Scale	Resistance and high-intensity interval training are effective in preventing the aggravation of cancer-related fatigue and decreasing the symptom burden.
Marx et al. [31]	2017	Not specified	Ginger (1,2 g orally)	FLIE-5DR INVR FACT-G FACIT-F	Ginger supplementation improved nausea, fatigue levels and overall general well-being in cancer patients.
Taaffe et al. [32]	2017	Prostate	Supervised impact loading and resistance exercises or Aerobic and resistance exercises	SF-36 EORTC QLQ-C36	Fatigue levels decreased in the group with impact loading and resistance exercises.
Kinkead et al. [33]	2017	Breast	Swedish massage therapy	Q-LES-Q MFI Promis Fatigue Short Form 7a	The intervention provided significant relief in cancer-related fatigue.
Wyatt et al. [34]	2016	Prostate	Saw palmetto	FACT-P	Saw palmetto can be a safe supplement for prostate cancer patients.
Do et al. [35]	2015	Breast	Aerobic, strengthening and stretching exercises	EORTC QLQ-C30 EORTC QLQ BR23 FSS	Patients undergoing this intervention showed improvements in quality of life, fatigue levels and physical symptoms.
Travier et al. [36]	2015	Breast	Aerobic and strength exercises or resistance exercises	MFI FQL SF-36 EORTC QLQ-30 Hospital Anxiety and Depression Scale	Exercises showed benefits in fatigue levels, muscle strength and cardiorespiratory fitness.
Gradalski et al. [37]	2015	Breast	Compressing bandage or Complex decongestive lymphatic training Physical-activity-assisted exercises	Lymphedema Questionnaire	Compressing bandages and physical exercises can be an effective treatment variant for limb lymphedema.
Han et al. [38]	2015	Lung	Chinese herbal medicine	FACT-L	The intervention proved to be well tolerated and could improve quality of life in lung cancer patients.
Courneya et al. [39]	2014	Breast	Aerobic exercises, resistance exercises	SF-36	Younger and slimmer breast cancer patients in premenopause undergoing chemotherapy were the most likely group to show improvements from a higher amount of exercise.
Rief et al. [40]	2014	Spinal bone metastases	Isometric resistance training or passive physical therapy, breathing exercises	EORTC QLQ-BM22 EORTC QRQ–FA13 FBK-R10	Isometric training can enhance the quality of life, reduce fatigue, and improve functional capacity.
Steindorf et al. [41]	2014	Breast	Progressive resistance training	EORTC QLQ-C30 EORTC QLQ BR23	The intervention ameliorated the quality of life and fatigue.
Schmidt et al. [42]	2014	Breast	Progressive resistance exercises	EORTC QLQ-C30 EORTC QLQ BR23	The study demonstrated benefits of resistance exercise over impact on fatigue and quality of life.
Pottel et al. [43]	2014	Head and Neck	Echium oil	PG-SGA EORTC QLQ-C30 EORTC QLQ- H&N35	The intervention proved to increase erythrocyte eicosapentanoic acid and gamma linolenic acid in patients suffering from head and neck cancer.
Ridner et al. [44]	2013	Breast	Low-level laser therapy	LSIDS-A BFI POMS-SF CES-D ULL-27 FACT-B	The therapy, alongside bandaging, can offer an alternative to manual lymphatic drainage.

Abbreviation: NRS Numerical Rating Scale; DASH Disabilities of the Arm, Shoulder, and Head; PQAS Pain Quality Assessment Scale; Global QoL Global Quality of life score; EORTC QLQ-C30 The European Organization for Research and Treatment of Cancer Quality of Life Questionnaire; MFI-20 multidimensional fatigue inventory; EORTC-QLQ-H&N35 The European Organization for Research and Treatment of Cancer Quality of Life Questionnaire -Head and neck module; FACT-ES Functional Assessment of Cancer Therapy-Endocrine Symptoms; FACT-B Functional Assessment of Cancer Therapy–Breast; BFI Brief Fatigue Inventory; SF 36 Medical Outcomes Survey Short Form 36 questionnaire; NCCN scale Fatigue Scale; EORTC QLQ-PAN26 The European Organization for Research and Treatment of Cancer Quality of Life Questionnaire Pancreatic-specific Module; EORTC QLQ-CIPN20 The European Organization for Research and Treatment of Cancer Quality of Life Questionnaire Chemotherapy-induced peripheral neuropathy module; CES-D Center for Epidemiological Studies Depression Scale; HADS Hospital Anxiety Depression Scale; POMS Profile of Mood States; MAC Measure of Adjustment to Cancer; UW-QOL University of Washington Quality of Life questionnaire; VAS Visual Analog Scale; FACT-F Functional Assessment of Cancer Therapy–Fatigue; EORTC QLQ-PR25 The European Organization for Research and Treatment of Cancer Quality of Life Questionnaire specific module for prostate cancer; FLIE-5DR Functional Living Index Emesis 5 Day Recall questionnaire; INVR The Rhodes Inventory of Nausea, Vomiting and Retching; FACT-G Functional Assessment of Cancer Therapy-Global Questionnaire; FACIT-F Functional Assessment of Chronic Illness Therapy-Fatigue Questionnaire; EORTC QLQ-C36 The European Organization for Research and Treatment of Cancer Quality of Life Questionnaire-Core 36; Q-LES-Q Quality of Life Enjoyment and Satisfaction Questionnaire; FACT-P Functional Assessment of Cancer Therapy-Prostate; EORTC QLQ-BR23 The European Organization for Research and Treatment of Cancer Quality of Life Questionnaire- Breast Cancer; FSS The Fatigue Severity Scale; FQL Fatigue Quality List; FACT-L Functional Assessment of Cancer Therapy-Lung; EORTC QLQ-BM22 The European Organization for Research and Treatment of Cancer Quality of Life Questionnaire—Bone metastases; EORTC QRQ–FA13 The European Organization for Research and Treatment of Cancer Quality of Life Questionnaire -Fatigue; FBK-R10 Effects of emotional distress questionnaire; PG-SGA Patient-Generated Subjective Global Assessment; LSIDS-A Lymphedema Symptom Intensity and Distress Scale-Arm; POMS-SF Profile of Mood States–Short Form; ULL-27 Upper Limb Lymphedema instrument.

## Data Availability

No new data were created or analyzed in this study.
